# Active video games and weight management in overweight children and adolescents—systematic review and meta-analysis

**DOI:** 10.1093/pubmed/fdad115

**Published:** 2023-07-26

**Authors:** M Bourke, L Patterson, F Di Nardo, P Whittaker, A Verma

**Affiliations:** Institute of Population Health, Manchester Academic Health Sciences Centre, University of Manchester, Manchester M13 9PT, UK; Institute of Population Health, Manchester Academic Health Sciences Centre, University of Manchester, Manchester M13 9PT, UK; Institute of Population Health, Manchester Academic Health Sciences Centre, University of Manchester, Manchester M13 9PT, UK; Institute of Public Health, Università Cattolica del Sacro Cuore, Rome, Italy; Institute of Population Health, Manchester Academic Health Sciences Centre, University of Manchester, Manchester M13 9PT, UK; Institute of Population Health, Manchester Academic Health Sciences Centre, University of Manchester, Manchester M13 9PT, UK

**Keywords:** children, health promotion, obesity

## Abstract

**Background:**

The prevalence of childhood obesity has been increasing for several decades. Active video games (AVG) may be an effective intervention to help manage this rising health crisis. The aim of this review is to evaluate whether AVG are effective at reducing weight or improving body composition in overweight youths.

**Method:**

Medline, Embase, SportDiscus, ASSIA, CINAHL Plus, CENTRAL, CDSR and PsychINFO databases were searched for studies assessing quantitative or qualitative impact of AVG in overweight adolescents published in English. Three authors screened the results using inclusion/exclusion criteria.

**Results:**

A total of 12 studies met the inclusion criteria; 11 reported a significant decrease in at least one weight outcome. Results from seven randomized controlled trials were pooled by meta-analysis, which compared with controls subjects in AVG groups demonstrated greater body mass index (BMI) Z-score reduction (mean difference: −0.09 (−0.12, −0.05) I2 = 34%, *P* < 0.0001). The mean weight reduction (−2.66 Kg (−5.67, +0.35) I2 = 0%, *P* = 0.08) and BMI (−2.29 (−4.81, +0.22) I2 = 49%, *P* = 0.07) were greater in AVG groups but results did not reach statistical significance.

**Conclusions:**

BMI Z-score was significantly reduced in the AVG group and the majority of included studies reported significant results in at least one weight outcome, suggesting AVG can be used to reduce weight or improve body composition in overweight youths. Further studies investigating the long-term sustainability of this change in body composition are needed.

## Introduction

The prevalence of childhood obesity has increased globally over several decades.^1^ Overweight children are more likely to be overweight adults.^2^ It is a risk factor for a range of diseases in later life including cancer, diabetes, cardiovascular disease and osteoarthritis.^3^^,^^4^ These diseases cause a decrease in quality of life, premature mortality and morbidity.^5^^,^^6^

Decreased levels of physical activity (PA), increased levels of sedentary time and an increased caloric intake are some of the factors influencing childhood obesity rates but these are not the only factors responsible.^7–10^ Screen-based activities increase children’s exposure to energy-dense food advertisements, leading to children consuming such food items.^11^ Reducing sedentary behaviours improves body composition in youths,^12^ and exercise is part of the management of paediatric obesity.^13–15^

Due to the popularity of video gaming, active videogames (AVG) may be an option to promote healthy living among children.^16^^,^^17^ Playing AVG results in an increased heart rate, oxygen consumption and energy expenditure.^18^ AVG may increase PA level sufficiently to produce healthy benefits in children and adolescents.^16^ Any increase in PA may produce positive healthy benefits.^19^ A lack of enjoyment is an indicator that children will not participate in exercise,^20^ therefore using AVG may overcome this barrier, as they stimulate enjoyment.^16^ Overweight children spend more time watching television and playing videogames than children who are not overweight.^21^

This study will aim to report on whether AVGs can be utilized, either solely or as part of a multi-faceted intervention, to reduce weight and improve body composition in overweight and obese youths.

## Methods

This review was conducted in accordance with the Preferred Reporting Items for Systematic Reviews and Meta-Analyses (PRISMA) criteria.^22^ This systematic review and meta-analysis was not registered with PROSPERO database.

### Literature search

In order to assess the effect of AVG on overweight children a systematic literature search was conducted by two researchers (LP, PW) on eight databases from the index date of each database through to October 2021. These databases were SportDiscus; ASSIA; Embase; Medline; CINAHL Plus; CENTRAL; CDSR and PsychINFO (Appendix 1). Inclusion and exclusion criteria are described in Table 1.

**Table 1:** Inclusion and exclusion criteria for the qualitative and quantitative analyses

Inclusion criteria for qualitative analysis	Exclusion criteria for qualitative analysis
Studies on obese/overweight children/adolescents Aim of the study is the assessment of exergaming Exergaming-based physical activity is actively encouraged in the study The study is published in English The study is a clinical trial	Neither weight or BMI are assessed as outcome Study population is exclusively made of subjects suffering from chronic diseases or in acute recovery
**Inclusion criteria for quantitative analysis**	**Exclusion criteria for quantitative analysis**
RCT	Subsets of data published in studies already included in this systematic review and meta-analysis

A ‘building block’ process (PICOS) was employed in constructing the search.^23^^,^^24^ Because AVG are a relatively recent phenomenon, none of the databases had a thesaurus heading for it. This intervention element was resolved by combining free text terms (using truncation and/or proximity operators) to find references to AVG. We used the combined thesaurus terms for obesity, paediatric obesity, overweight and body mass index (BMI). We conformed to the World Health Organization (WHO) definition of age range for adolescents (10–19).^25^ This term was combined with other thesaurus terms for children, excluding infants, to encompass an age range of 2–19. Three researchers (MB, LP, PW) independently screened the retrieved papers by titles and abstracts and the eligible studies were further screened by full text. The search was supplemented with reference and citation tracking of studies included in the qualitative analysis.^26^

Only studies that were published in English were included. Studies that were not randomized were included in the qualitative but not the quantitative analysis. Studies were first selected if the inclusion criteria were met for the qualitative analysis and further criteria were then applied to these studies to determine which would be selected for the quantitative analysis.

### Quality assessment

The quality of the studies included was assessed using the Public Health Critical Appraisal Checklist.^27^ The checklist consists of 23 criteria, including whether the study design, sampling and data collection was appropriate; whether confounders were considered; whether the study was ethical; whether the statistics were appropriate; and whether the results of the study are relevant to public health practice. Each study was assessed against the check list,^27^ and was determined to be of high, moderate or low quality depending on how many criteria the study satisfactorily fulfilled on the checklist.^27^ The more the criteria that were satisfactorily fulfilled, the higher the quality of research study was deemed to be. The quality of the included studies was assessed by two authors independently and a consensus was reached where there was disagreement.

### Qualitative analysis

A narrative summary of the main findings of the papers included in the qualitative analysis is provided.

### Quantitative analysis

All randomized controlled trials (RCTs) comparing exergaming with controls in terms of weight, BMI, BMI Z-scores (or BMI percentiles) and/or body mass composition (percentage or fat and/or lean mass) were included in the quantitative analysis. BMI percentiles, where present, were converted into BMI Z-scores. Subsets of data published in studies already included in this systematic review and meta-analysis were excluded. Interventions in the control groups were either nothing or recommendations on PA or other schemes of non-exergaming PA. Where more than two groups of exergaming PA were present in the same study, their data were merged into one intervention group for the purposes of the comparison with controls.

Two investigators (FDN, LP) extracted data independently. Disagreements were resolved by consensus after contacting the authors of the included studies.

Where three or more studies reported the same outcome, a meta-analysis examining pooled effect estimates with 95% confidence intervals (CI) was performed using both fixed and random effects models and using the random effects model in interpretation of findings. Heterogeneity was assessed with the I^2^ index. The overall effect was tested using Z-scores and statistical significance was set at *P* < 0.05. Where possible, subgroup analyses were performed using data from intention-to-treat studies only.

Data analyses were performed using Review Manager (RevMan) version 5.3.^28^

## Results

**Fig. 1:**
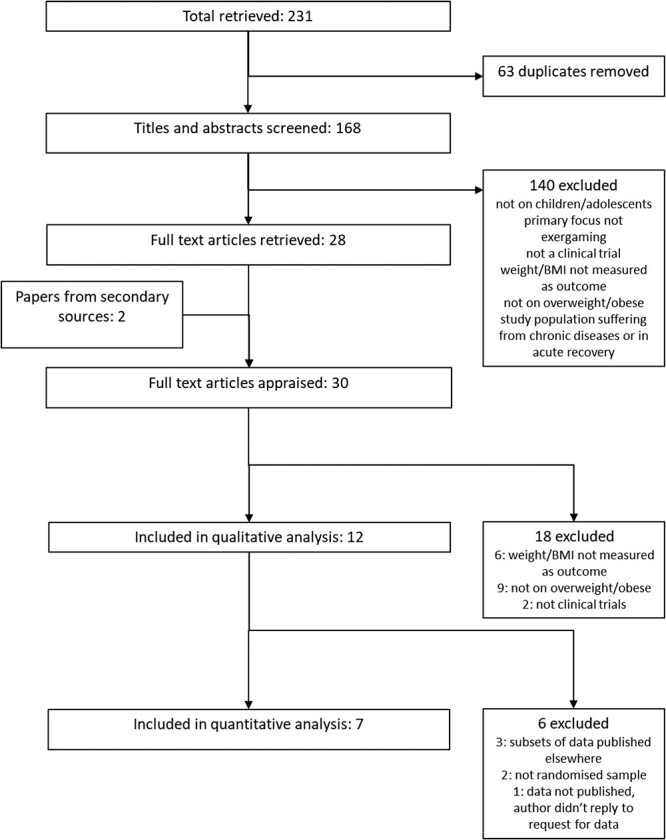
PRISMA flow chart.

A total of 168 unique articles’ titles and abstracts were screened. A consensus was reached on 30 articles for full-text appraisal; 12 articles entered the qualitative analysis, seven entered the quantitative analysis (Fig. 1).

A total of 10 individual interventions reported in 12 studies were included (Table 2);^29–40^ 10 studies were RCT^29^^,^^32–40^ and two were pilot studies.^30^^,^^31^ The sample sizes varied between 4 and 327, with the ages between 7 and 19 years. The studies were conducted in Canada,^29^ Brazil,^30^ USA,^31^^,^^36–40^ Iran^33^ and New Zealand.^32^^,^^34^^,^^35^ The length of the interventions varied between 6 and 24 weeks. The two pilot studies consisted of only one intervention group each with no control group.^30^^,^^31^ Nine of the RCTs used a control with no intervention.^32–40^ Four studies were conducted in a lab.^29^^,^^30^^,^^31^^,^^40^ One study conducted the active video gaming within the community but in a facilitated setting.^31^ Five studies provided the participants with the AVG to take home.^31^^,^^33–36^ One study was conducted within a school.^38^

**Table 2:** Summary of included studies

1st author	Year	Country	Aim	Population & Setting	Intervention	Comparator	Outcomes	Main finding	Advantages	Limitations	Quality
Adamo et al.^29^	2010	Canada	To compare the effects of a cycling active video game using the GameBike™ with cycling to music on adherence, duration, intensity of exercise, aerobic fitness, metabolic parameters, body composition & image, perceived scholastic ability, social acceptance, athletic competence and global self-esteem using RCT design in overweight & obese teens. Associations between changes in psychosocial functioning with changes in fitness and body composition were also compared.	*n* = 30 Families screened through the endocrine clinic at the Children’s Hospital of Eastern Ontario	Participants were required to exercise on a GameBike™ active video game for two 60 min lab sessions per week for 10 weeks. Participants had to stay in the lab for the full 60 min but were allowed breaks. Study lasted 10 weeks.	Cycling on the GameBike™ to music without using the video game	Weight (Kg), BMI, BMI percentile, waist circumference, body fat %, fat mass, fat-free mass, psychosocial functioning, body image	A significant effect of a decrease in body fat percentage when the two groups were combined was found. No significant difference between exercise groups but improvements found when groups combined.	Comparator of same exercise, w/o active video game, removed dietary confounder, objectively measured weight outcomes	Small sample size; lack of non- exercise control; participants who completed intervention may be more motivated, poor generalizability, subjective academic performance measurements	Moderate
Carrasco et al.^30^	2013	Brazil	A pilot study to assess the effects of active gaming on the functional capacity and body composition of overweight children using exercises on the Nintendo® Wii console.	*n* = 4 The study was conducted at a physio-therapy clinic. Subjects were recruited from schools.	Exercises were performed with the Nintendo® Wii using aerobic activities such as running, boxing and karate starting at lower intensities and increasing each week for 6 weeks.	None	Body mass (Kg), BMI, abdominal circumference, BMI percentile	A significant decrease in BMI, body mass and abdominal circumference	Significant effect despite intervention is a pilot study	Very small sample; no control; very obese children therefore would have been significant	Low
Christison et al.^32^	2012	USA	To demonstrate the effectiveness of a community-based weight management program that involved active video gaming via a pilot study.	*n* = 48 Participants were referred from primary care practice or self-referred	A community-based weight management program comprising a curriculum of exercise class, nutritional education, behavioural modification education and exergaming, which lasted for 10 weeks.	None	Weight (Kg), BMI, BMI Z-score	A significant decrease in BMI and BMI Z-score, with very little change in overall weight	Large number of subjects for a pilot study	Small sample; no control; the effect of adding an active video game to the curriculum used cannot be truly assessed	Moderate
Irandoust et al.^33^	2020	Iran	The objective of this RCT was to compare the effects of exergames with digital games or aquatic sports on weight outcomes in overweight male children against a control group	*n* = 61 Participants were invited to the study from a primary school	Video gaming group played 3x 60 min sessions per week of Xbox Kinect over 12 weeks. Aerobic aquatic group performed 3x 60 min sessions per week over 12 weeks.	Control group	Weight (Kg), weight Z-score, BMI, BMI percentile, BMI Z-score	Exergames and aquatic exercises are beneficial for improving body composition of overweight children	Randomization; control; significant results despite small sample	Small sample sizes from a single school; only male children included; intervention within a lab setting	Moderate
Maddison et al.^34^ Maddison et al.^35^ Foley et al.^32^	2011 2012 2014	New Zealand	The objective of this RCT was to assess the effect of AVG on weight, body composition, PA and physical fitness. A subgroup analysis to examine the trail effects by ethnicity, sex and baseline cardiovascular fitness was conducted. Mediators of the effects of the intervention on body composition were investigated.	*n* = 327 Children were recruited from various places	Children were encouraged to meet PA recommendations by supplementing periods of inactivity with active video game play. The study ran for 24 weeks.	Control group	Weight (Kg), BMI, BMI Z-score, waist circumference, body fat %, fat mass, fat-free mass	A significant treatment effect was shown for the change from baseline in BMI & BMI Z-score favouring the intervention group. There was no evidence of treatment differences among the subgroups. The positive effect on body composition is most likely mediated through aerobic fitness.	Large sample; control; real-life setting; long follow-up; sample calculation; sufficient power	Assessments were not blinded; snack food/videogames diaries were developed & piloted for this study; % body fat was below optimal accuracy	High High High
Murphy et al.^36^	2013	USA	The objective of the RCT was to determine the effectiveness of playing Dance Dance Revolution® on endothelial dysfunction and other risk factors in overweight children.	*n* = 35 Subjects were recruited from a larger study	Subjects were encouraged to use Dance Dance Revolution® 5 days/week for a set amount of time that increase every week until the 5^th^ week for 12 weeks.	Control group	Weight (Kg), BMI	The exercise group gained significantly less weight than the control group	Control group	Lack of information about randomization; small sample; multiple univariate analysis used and no Bonferroni correction therefore type 1 error cannot be ruled out	Low
Staiano et al.^37^	2017	USA	The aim of this RCT is to assess the effectiveness of an exergame intervention on weight outcomes of overweight adolescents.	*n* = 46 Participants were recruited through different forms of advertisement	Participants were encouraged to play active video games for 60 min every day over 24 weeks. Curriculum was provided with instructions on increasing intensity and challenge each week.	Maintain normal level of physical activity over the 24 week period.	BMI Z-score, weight Z-score, fat mass percentage	The exergame intervention reduced BMI Z-score and improved cardio-metabolic health among overweight children	Control group; randomization; power calculation performed	Small sample size; relied on gaming adherence relied on child and parental self-report	High
Staiano et al.^38^	2013	USA	This RCT examined whether a 20 week exergame intervention can produce weight loss and improve psychosocial outcomes for overweight African American adolescents and the impact of playing cooperative or competitively.	*n* = 54 Participants were recruited from a high school and referral from the school wellness clinic.	Participants in exergame conditions could play the Nintendo® Wii active exergame 30–60 min every school day during lunch period or after school, for 20 weeks.	Cooperative, competitive and control groups.	BMI percentile	The cooperative group lost significantly more weight than the control.	Control group	Small sample from a single group; lack of weight outcomes; no explanation of randomization; high participant attrition; lack of generalizability	Low
Trost et al.^39^	2014	USA	The aim of this RCT was to report the effects of using active video gaming in a pediatric weight management program.	*n* = 75 Participants were recruited via various means	All participants were enrolled in a family-based pediatric weight management program. In addition, participants randomized to active video game group were provided with equipment and games. No explicit advice or goals were given. Program last 16 weeks.	Program and active gaming intervention or program-only intervention	BMI Z-score	Both groups exhibited significant reductions in BMI Z-score. Subjects in the active gaming group exhibit a significantly greater reduction in BMI Z-score.	Comparator group; intention to treat analysis calculated; can be generalized to other populations	Lack of weight outcomes; missing data significant as many participants did not deliver valid monitoring days; no formal cost effectiveness analysis	High
Wagener et al.^40^	2012	USA	To investigate the impact of dance-based exergaming on a diverse sample of obese adolescents’ perceived competence to exercise, psychological adjustment and BMI in an RCT.	*n* = 41 Adolescents were assessed for eligibility	Supervised 10-week group-dance-based exergame exercise program. Adolescents returned to the clinic 2–3 per week for 40 min sessions.	Control group	BMI Z-score, self-reported competency regarding exercise	Findings indicate a positive effect on exergaming on psychological adjustment and perceived competence to exercise. No changes in BMI Z-scores were noted.	Control, ethnically diverse sample, blinded study, objectively measured weight outcomes	Small sample size, lack of long-term follow-up and measurement of participant exercise outside of the trial and reduced external validity given the laboratory setting	Moderate

### Quality assessment

Using the Public Health Critical Appraisal Checklist,^27^ five studies were of high quality^32^^,^^34^^,^^35^^,^^37^^,^^39^; four studies of middle quality^29^^,^^31^^,^^33^^,^^40^ and three studies of low quality.^30^^,^^36^^,^^38^

Four studies do not provide information on what equipment they used to measure the height and weight.^31^^,^^36^^,^^38^^,^^39^ The other studies all used stadiometers to measure height^29^^,^^30^^,^^32–35^^,^^37^^,^^40^ and measured weight using bioelectrical impedance scales,^29^^,^^33^ calibrated scales,^30^ Salter scales,^32^^,^^34^^,^^35^ digital scales^37^ and calibrated electronic scales.^39^

### Qualitative analysis

#### Characteristics of the included studies are summarized in Table 2

Analysed outcomes were weight (8 out of 12 studies),^29–36^ BMI (8/12), ^29–36^ BMI Z-scores (8/12),^31–35^^,^^37^^,^^39^^,^^40^ waist circumference (4/12),^29^^,^^32^^,^^34^^,^^35^ body fat percentage (5/12),,^29^^,^  ^30^^,^^32^^,^^34^^,^^35^ fat mass (4/12),^29^^,^^32^^,^^34^^,^^35^ BMI percentile (4/12),^29^^,^^30^^,^^33^^,^^38^ fat-free mass (3/12),^32^^,^^34^^,^^35^ weight Z-score (2/12),^33^^,^^37^ abdominal circumference (1/12)^30^ and fat mass percentile (1/12).^37^

A total of 11 studies (11/12) reported a significant decrease in at least one weight outcome.^29–39^ One study reported a significant decrease in body fat percentage but only when two intervention groups, cycling to AVG or music, were combined.^29^ Three interventions reported a significant decrease in BMI.^30–35^ Four interventions demonstrated a significant reduction in BMI Z-scores^31^^,^^32^^,^^34^^,^^35^^,^^37^^,^^39^ and one did not.^40^ In one study, a significant reduction was only observed after an outlier had been removed from the control group who had decreased their BMI Z-score by 3.3 standard deviations below the mean.^37^ The cooperative group of the intervention by Staiano *et al*.^38^ described a significant drop in BMI percentile compared with the control, though this was not mirrored in the competitive arm. One study reported a significant decrease in abdominal circumference.^30^ Several interventions recorded a significant decline in body fat percentage^29^^,^^32^^,^^34^^,^^35^ and significant decrease in fat mass.^32^^,^^34^^,^^35^

Certain interventions consisted of an AVG only intervention and control (7/12). Maddison *et al*.^34^ reported a significant reduced BMI (0.24) (*P* = 0.02), BMI Z-score (0.06) (*P* = 0.03), weight (0.72 kg) (*P* = 0.02), body fat percentage (0.83%) (*P* = 0.02) and body fat (0.8 kg) (*P* = 0.05). No significant differences were identified when a sub-group analysis was performed between ethnicity and sex.^32^ Irandoust *et al*.^33^ report a significant decrease in body weight and BMI. Murphy *et al*.^36^ described a significantly smaller weight gain in the intervention group of 0.91 pounds compared with an increase of 2.43 pounds in the control group (*P* = 0.017). There was a non-significant change in BMI. Staiano *et al*. reported a significant decrease in weight Z-score in both the intent-to-treat intervention group of −0.1 (standard deviation 0.05) versus control 0.04 (standard deviation 0.05) (*P* = 0.049) and without the outlier (intervention −0.09 (0.05) versus control 0.07 (0.04) (*P* = 0.022)). There was no significant difference for BMI Z-score with the intent to treat analysis; however, without the outlier, there was a significant change (intervention −0.06 (0.03) versus control 0.03 (0.03) (*P* = 0.016)).^36^ Wagener *et al*.^39^ described no significant change in BMI Z-score.

Carrasco *et al*.^30^ describe a significant decrease in body mass (47 to 45 kg) (*P* = 0.0018), BMI (23.02 to 22.22) (*P* = 0.0005) and abdominal circumference (81.82 to 79.97 cm) (*P* = 0.0223). Christison *et al.*^31^ described a study with a significant decrease in BMI (31.07 to 30.59) (*P* = 0.002) and BMI Z-score (2.24 to 2.17) (*P* < 0.0001).

Staiano *et al*.^38^ describe a study consisting of two similar intervention groups (cooperative and competitive) and a control group. The cooperative group experienced a significantly greater weight loss than the control (mean = 1.65 kg, *P* = 0.021) with the BMI percentile decreasing from 93.93% to 84.74%. There was no significant difference in weight between the competitive group and the control.

Adamo *et al.*^29^ compared cycling with an AVG and cycling to music. There were no significant changes on body weight, BMI, fat mass, free fat mass and waist circumference. There was a small significant reduction in body fat percentage when the two groups were combined.

Trost *et al.*^39^ compared two groups which consisted of a weight management program, one with AVG and one without. The AVG group experienced a greater reduction in BMI Z-score (0.14) (*P* < 0.001).

### Quantitative analysis

#### Seven studies entered the quantitative analysis (Fig. 1) ^29^^,^  ^34^^,^  ^36–40^

Table 3 shows the main characteristics of the RCTs that entered the quantitative analysis and results of the data extraction. Four RCTs, accounting for 358 randomized subjects, reported data on weight,^29^^,^^34^^,^^36^^,^^38^ three, accounting for 319 individuals, reported data on BMI^29^^,^^34^^,^^36^ and five, accounting for 433 randomized subjects, reported BMI Z-scores allowing calculation of pooled effects estimates.^29^^,^^34^^,^^37^^,^^39^^,^^40^

**Table 3:** Summary of the characteristics of the randomized clinical trials that entered the quantitative analysis and results of the data extraction

	Characteristics of the randomized clinical trials included in the meta-analysis					Pop. size		BMI Z-score		BMI		Weight		W. Circ.		Fat mass (%)	
Study, year	Characteristics of the population	Duration (days) and frequency	Intervention	Control	Attendance and data type	EXG	CNT	EXG	CNT	EXG	CNT	EXG	CNT	EXG	CNT	EXG	CNT
Adamo et al.^29^	BMI over 95^th^ % or uncontrolled blood glucose and BMI over 85^th^ %; Age range: 12–17, Mean 14.5 (1.6)	70 (twice a week)	Interactive video game cycling and a selection of compatible PS2 videogames	Cycling with music	Required (reimbursed); Intention-to-treat	13	13	1.96 (0.2)	2.01 (0.5)	35.5 (9.7)	39.4 (8.9)	93.7 (21.0)	106.8 (24.2)	111.3 (16.1)	115.6 (15.5)	43.5 (7.8)	42.1 (11.7)
Maddison et al.^34^	Overweight or obese; Age range: 10–14, Mean: 11.6 (1.1)	168 (any day)	EyeToy, dancemat and a selection of active PS3 videogames	Nothing	Recommended; Intention-to-treat	123	135	1.1 (1.1)	1.3 (1.0)	24.8 (3.6)	25.8 (4.2)	63.0 (12.3)	64.8 (14.4)	84.4 (10.8)	88.0 (10.7)	29.8 (7.2)	31.1 (6.3)
Murphy et al.^36^	BMI over 85^th^ %; Age range: 12–17	84 (5 days a week)	Dance Dance Revolution on PS2	Nothing	Encouraged	23	12	N/A	N/A	27.8 (4.7)	32.1 (4.9)	63.4 (15.5)	71.9 (16.6)	N/A	N/A	N/A	N/A
					(no incentives); Intention-to-treat												
Staiano et al.^37^	BMI over or equal to 85^th^ %; Age 10–12	168 (60 min per day)	Xbox 360 & Kinect with a selection of games	Normal level of physical activity for participant	Encouraged – by coaches and advised to play with friends or family; Intention-to-treat	22	23	2.00 (0.03)	2.12 (0.03)	N/A	N/A	N/A	N/A	N/A	N/A	41.5 (0.4)	43.8 (0.4)
Staiano et al.^38^	BMI over 75^th^ %; Age range: 15–19	135 (every school day)	Cooperative or competitive Nintendo Wii active game	Nothing	Encouraged (with incentives);	27	12	N/A	N/A	N/A	N/A	91.3 (18.7)	94.2 (20.9)	N/A	N/A	N/A	N/A
					Per protocol												
Trost et al.^39^	BMI over 85^th^ %; Mean age: 10 (1.7)	112 (3 or more valid monitoring days)	Behavioural treatment and Xbox Kinect plus one sports game	Behavioural treatment	No specific attendance goals; Intention-to-treat	28	35	1.95 (0.08)	2.09 (0.07)	N/A	N/A	N/A	N/A	N/A	N/A	N/A	N/A
Wagener et al.^40^	BMI over 95^th^ %; Age range: 12–18 Mean: 14 (1.7)	70 (3 times a week)	Dance-based exergaming (arcade)	Nothing	Required (reimbursed);	21	20	3.13 (0.19)	3.12 (0.20)	N/A	N/A	N/A	N/A	N/A	N/A	N/A	N/A
					Per protocol												

**Fig. 2:**
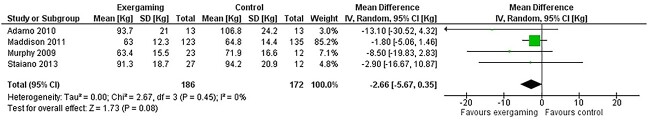
Weight (Kg). Pooled mean difference. Forest plots of the observed outcomes (random model) and 95% CI.

**Fig. 3:**
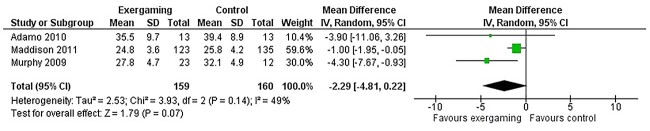
BMI. Pooled mean difference. Forest plots of the observed outcomes (random model) and 95% CI.

**Fig. 4:**
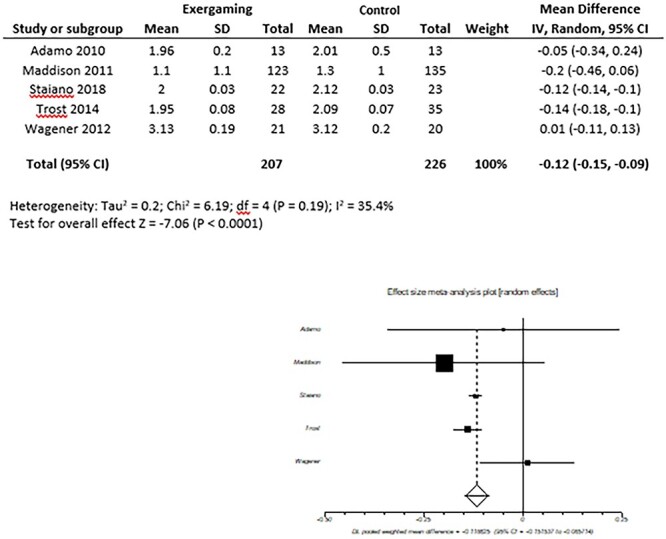
BMI Z-score. Pooled mean difference. Forest plots of the observed outcomes (random model) and 95% CI.

Subjects who underwent AVG were more likely to show lower weight compared with the control groups (mean difference [random effect]: −2.66 Kg; 95%CI: −5.67, +0.35), with no heterogeneity (I^2^ = 0%). These differences were not statistically significant (*P* = 0.08, Fig. 2) and this was still true after selecting intention-to-treat studies only. Participants in the exergaming groups also showed lower BMI values (mean difference [random effect]: -2.29; 95%CI: −4.81, +0.22) with high heterogeneity (I^2^ = 49%) and no significant results (*P* = 0.07, Fig. 3). This is possibly due to heterogeneity, as the same outcome showed significant results using the fixed effect model (−1.29; 95%CI: −2.20, −0.38; *P* = 0.006). BMI Z-score was significantly reduced among the exergaming population (mean difference: −0.09; 95%CI: −0.12, −0.05) but, again, with high heterogeneity (I^2^ = 34%) and statistically significant results (*P* < 0.0001, Fig. 4).

## Discussion

### Main findings of this study

A total of 11 studies reported significant results in at least one weight outcome. A significantly lower BMI Z-score was observed within the meta-analysis.

Four interventions measuring BMI^30^^,^^31^^,^^33^^,^^34^ and four interventions calculating BMI Z-scores^31^^,^^34^^,^^37^^,^^39^ resulted in a significant decrease from baseline. Similar results have been found in a comparable intervention carried out in children of mixed weights.^41^

The results of meta-analysis are likely due to high heterogeneity, small samples and small number of available RCTs. Heterogeneity can be explained by the inconsistent methodologies in these studies: the interventions varied, and even the controls. Only a limited number of subgroup analyses were possible due to the low number of included studies. Due to the low number of studies included in the meta-analysis, it was not possible to produce funnel plots and analyse for publication bias.

### What is already known on this topic

Exergaming technologies are relatively new and have not been considered as a viable weight control or PA promotional methodology by many researchers. AVG have been shown to elicit a higher energy expenditure in children compared with sedentary activities and have shown to increase heart rate, oxygen consumption and energy expenditure, similar to that of light to moderate PA in children.^18^^,^^42^^,^^43^ However, it is less well known whether children using AVG as exercise would play with sufficient vigor and frequency to gain cardiovascular or health benefits.^43^

The enjoyment that AVG are planned to stimulate may be essential to their ability to promote PA and thereby help children manage their weight more healthily. Boredom has been shown to be a barrier to long-term AVG play.^16^ Longer interventions in this review experienced higher dropout rates.^34^^,^^38^ One potential option of overcoming the barrier of boredom of AVG play after prolonged use is to use multiple games.^31–35^^,^^37^^,^^39^

### What this study adds

This systematic review and meta-analysis investigated the effectiveness of AVG to aid in the weight management of overweight children and adolescents. The results suggest that AVG can help reduce the increasing trend of childhood obesity, either solely or within a well-established weight management program.

Exergaming can be utilized as one component in a multi-focal weight management intervention or as the sole constituent. Many interventions employed AVG as the sole component.^29^^,^^30^^,^^32–38^^,^^40^ Two incorporated AVG into a holistic weight management program.^31^^,^^39^ Combining a weight management program with AVG can improve the health outcomes compared with a more traditional weight management program.^39^ This may be because children playing AVG participated in more exercise compared with children who did not.^39^ Another intervention demonstrated similar results.^31^ It has been suggested that a holistic approach is needed for public health interventions to be successful.^44^ Therefore, it may be beneficial to combine AVG with interventions that target other behavioural changes.

AVG-only interventions have been shown to cause a decrease in weight outcomes in overweight children^29^^,^^30^^,^^32–39^ and this may be because children are replacing sedentary behaviours with PA.^19^ A weight management intervention which included AVG resulted in children decreasing their non-AVG play by ~9 min/day and increasing their AVG play by 10 min/day compared with controls.^34^ This may minimize calorific intake by reducing exposure to snack food advertisement.^45^

Children increase in weight as they’re growing. The best outcome to evaluate weight control is therefore the BMI Z-score.^46^ Body mass composition could be taken into consideration, but its distribution in the population is affected by gender and age (normal fat mass percentages are higher in females and change over time).

Interventions in all settings produced significant findings towards a healthier trend. It may be advantageous to run AVG interventions from children’s homes as it may be easier for parents to encourage their children to play AVG rather than encouraging them to abstain from videogames altogether.^34^

Researchers may want to cooperate with the videogame industry to produce games that aim to control weight and achieve the recommended PA levels in such a way that the activity is enjoyable and sustainable. All games examined in these trials were created with the purpose of entertaining a broad audience. However, if they were designed with the express aim of producing positive effects on the health of children and adolescents while incorporating the principles of the evidence-based medicine, then perhaps we could observe more robust results.

Future studies should focus on interventions with bigger sample sizes and longer follow-up period to observe if AVG can result in a prolonged change in body composition in overweight youths.

### Limitations of this study

Limitations of the individual studies include small sample sizes, high dropout rates, lack of long-term follow-up and low number of weight outcomes measured. One study was limited as the children were very overweight and therefore most interventions would be more likely to have a positive significant effect.^30^ Only three interventions had a follow-up period that lasted several months and all of these had the highest dropout rates.^33^^,^^34^^,^^38^ The follow-up period in these studies is possibly still not sufficient to determine the long-term effects of AVG on weight outcomes of overweight children.

Out of the 12 studies included in the qualitative analysis, only two^30^^,^^32^ are based in low-/middle-income countries and none of the studies included children below the age of 7. This could limit the generalizability of the results of this review to older children from higher income countries.

Grey literature was excluded from the search since it was found to be seriously affected by the marketable nature of the products examined. Literature with commercial purposes tends to highlight the positive features ignoring potential adverse effects for health, while the media sometimes exaggerates the negative effects and can even exhibit hostility to videogames.

However, strengths of this study must also be considered. This is one of the first studies to summarize the literature on AVG use in weight loss in overweight youths and quantify the effectiveness of interventions using a meta-analysis. AVG are a recent development and therefore our study has looked at a novel tool that is designed to stimulate interest, maintain engagement and has the potential to shift some screen time from sedentary to active, to aid in weight loss and improve body composition in overweight children and teenagers.

## Conclusions

Although only BMI Z-score was significantly reduced in the AVG group, results are still promising, as 11 of 12 studies reported at least one significantly improved weight outcome, suggesting that more RCTs with standardized methodology, bigger samples, intention-to-treat protocols, longer follow-up, children and teenagers from all age groups and assessment of BMI Z-score and body mass composition could find beneficial effects of AVG on weight control in overweight children and adolescents. Such studies are thus encouraged. Videogame industry and researchers could cooperate to produce evidence-based exergaming strategies that are suited for children and adolescents and aim at controlling weight and achieving internationally recommended PA levels.

## Supplementary Material

Appendices_fdad115

## Abbreviations

PA, physical activity; AVG, active videogames; WHO, World Health Organization; BMI, body mass index; RCT, randomized controlled trials

## Notes

**M. Bourke,** Paediatric Doctor

**L. Patterson,** Statistician

**F. Di Nardo,** Statistician

**P. Whittaker,** Public Health Doctor

**A. Verma,** Public Health Doctor
