# Supplementary material for: Active video games and weight management in overweight children and adolescents—systematic review and meta-analysis
Source: J Public Health (Oxf). 2023 Jul 26;45(4):935–46. doi: 10.1093/pubmed/fdad115 (PMC10788842; doi:10.1093/pubmed/fdad115)

***Appendix 1: Search strategy for 8 databases***

**EMBASE**


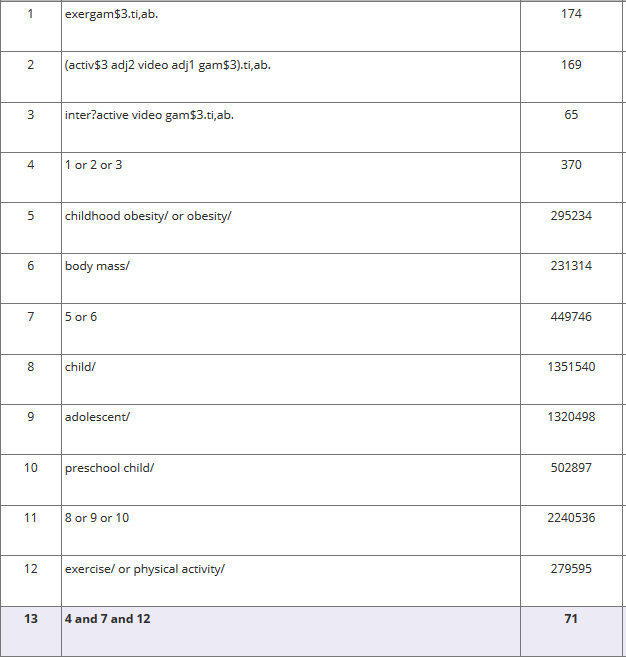


**MEDLINE**


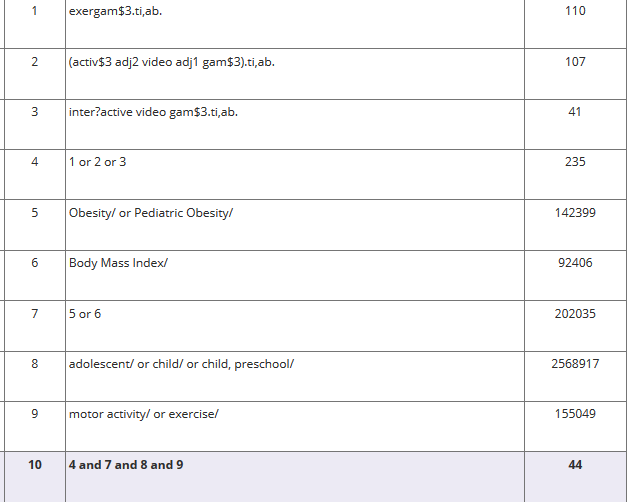


**PsychINFO**


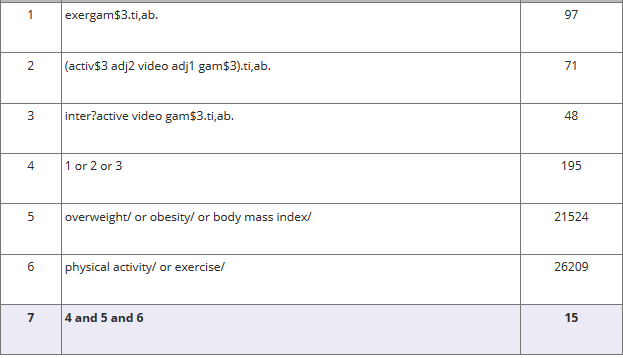


**Cochrane CENTRAL & CDSR**


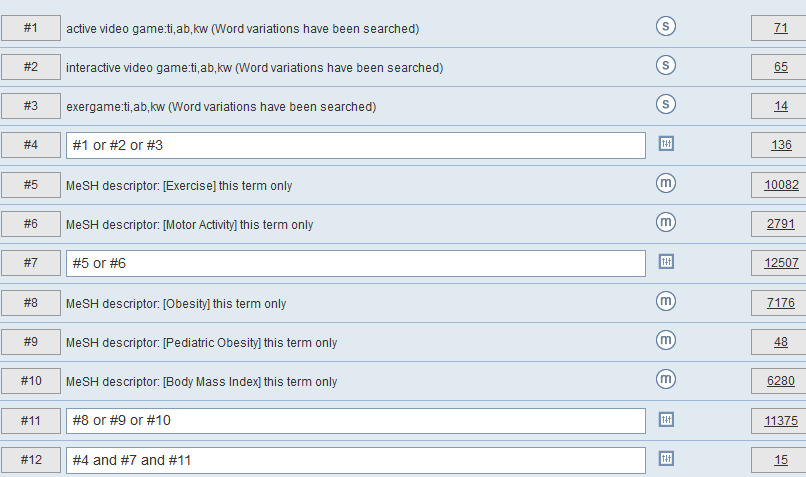


**SportDiscus**


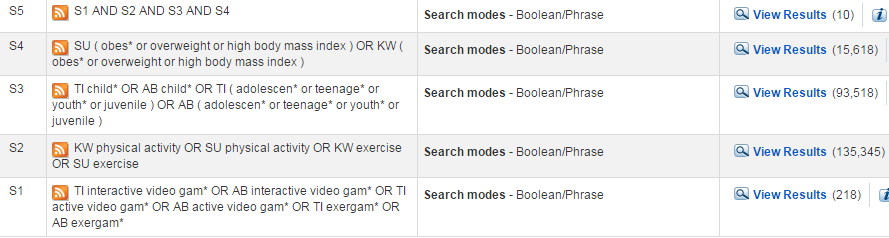


**CINAHL Plus**


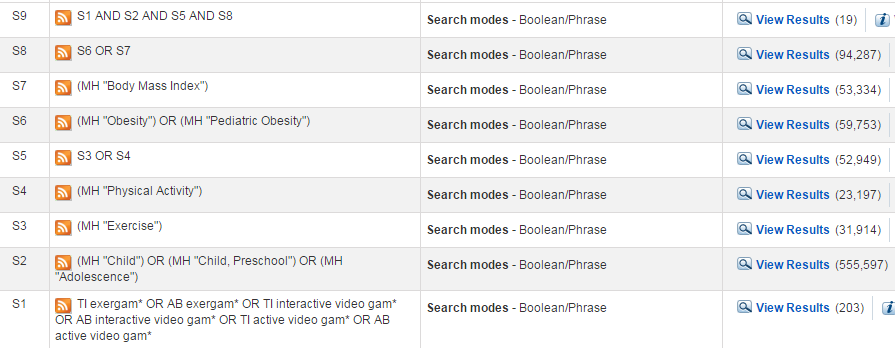


**ASSIA**


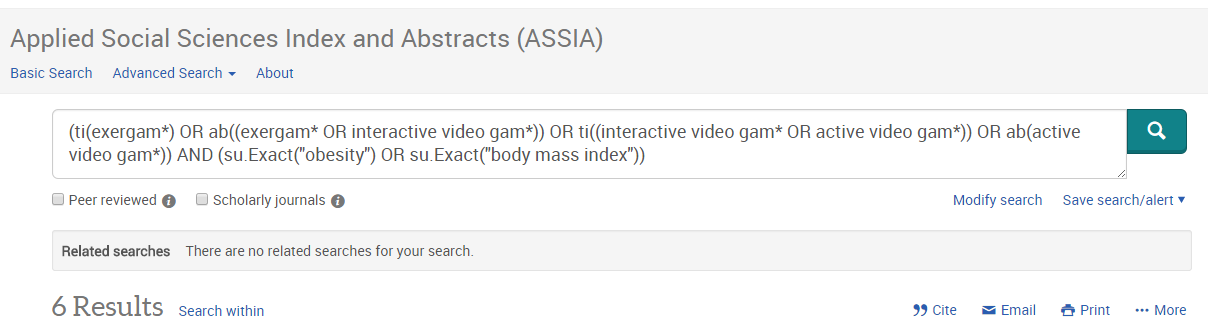


***Appendix 2: Public Health Critical Appraisal Checklist***


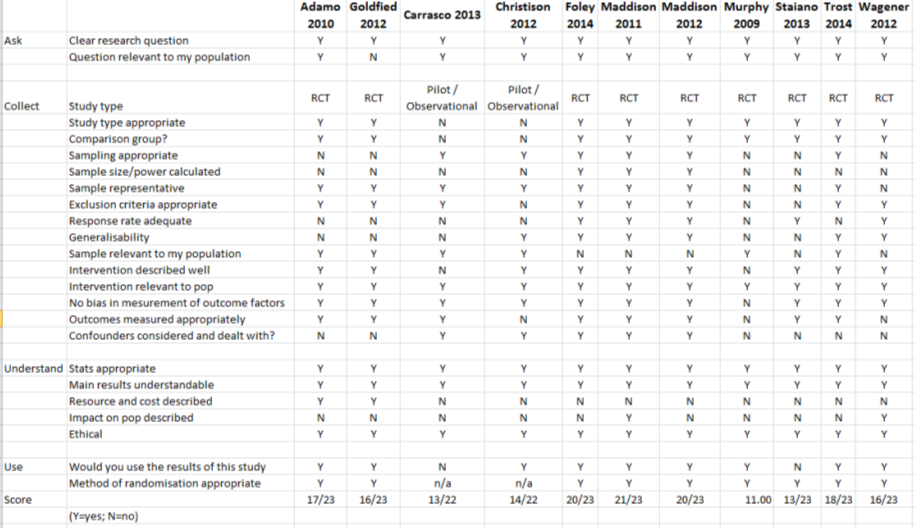

Supplement: Appendices_fdad115 [file appendices_fdad115.docx]
